# A trans fatty acid substitute enhanced development of liver proliferative lesions induced in mice by feeding a choline-deficient, methionine-lowered, L-amino acid-defined, high-fat diet

**DOI:** 10.1186/s12944-020-01423-3

**Published:** 2020-12-14

**Authors:** Noriko Suzuki-Kemuriyama, Akari Abe, Kinuko Uno, Shuji Ogawa, Atsushi Watanabe, Ryuhei Sano, Megumi Yuki, Katsuhiro Miyajima, Dai Nakae

**Affiliations:** 1 Department of Nutritional Science and Food Safety, Faculty of Applied Bioscience, Tokyo University of Agriculture , 1-1-1, Sakuragaoka, Setagaya, Tokyo, 156-8502 Japan; 2 Department of Nutritional Science and Food Safety, Graduate School of Applied Bioscience, Tokyo University of Agriculture, 1-1-1 Sakuragaoka, Setagaya, Tokyo, 156-8502 Japan; 3 Department of Food and Nutritional Science, Graduate School of Applied Bioscience, Tokyo University of Agriculture, 1-1-1, Sakuragaoka, Setagaya, Tokyo, 156-8502 Japan

**Keywords:** Nonalcoholic steatohepatitis, *Trans* fatty acid substitutes, Choline-deficient, Methionine-lowered, L-amino acid-defined, High-fat diet

## Abstract

**Background:**

Nonalcoholic steatohepatitis (NASH) is a form of liver disease characterized by steatosis, necroinflammation, and fibrosis, resulting in cirrhosis and cancer. Efforts have focused on reducing the intake of *trans* fatty acids (TFAs) because of potential hazards to human health and the increased risk for NASH. However, the health benefits of reducing dietary TFAs have not been fully elucidated. Here, the effects of TFAs vs. a substitute on NASH induced in mice by feeding a choline-deficient, methionine-lowered, L-amino acid-defined, high-fat diet (CDAA-HF) were investigated.

**Methods:**

Mice were fed CDAA-HF containing shortening with TFAs (CDAA-HF-T(+)), CDAA-HF containing shortening without TFAs (CDAA-HF-T(−)), or a control chow for 13 or 26 weeks.

**Results:**

At week 13, NASH was induced in mice by feeding CDAA-HF-T(+) containing TFAs or CDAA-HF-T(−) containing no TFAs, but rather mostly saturated fatty acids (FAs), as evidenced by elevated serum transaminase activity and liver changes, including steatosis, inflammation, and fibrosis. CDAA-HF-T(−) induced a greater extent of hepatocellular apoptosis at week 13. At week 26, proliferative (preneoplastic and non-neoplastic) nodular lesions were more pronounced in mice fed CDAA-HF-T(−) than CDAA-HF-T(+).

**Conclusions:**

Replacement of dietary TFAs with a substitute promoted the development of proliferation lesions in the liver of a mouse NASH model, at least under the present conditions. Attention should be paid regarding use of TFA substitutes in foods for human consumption, and a balance of FAs is likely more important than the particular types of FAs.

**Supplementary Information:**

The online version contains supplementary material available at 10.1186/s12944-020-01423-3.

## Background

Nonalcoholic steatohepatitis (NASH) is a form of liver disease characterized by steatosis, inflammation, ballooning, and subsequent death of hepatocytes. It is also associated with hepatic fibrosis, resulting in cirrhosis and cancer [1–3]. It has been hypothesized that the development of NASH requires two “hits” [4]: the first hit represents the development of hepatic steatosis, and the second hit involves oxidative stress and proinflammatory cytokines, inducing further liver injury. Inflammation activates a stress response, resulting in a wide variety of injuries to hepatocytes, including lipidosis, resulting in hepatocellular lipid accumulation (i.e., steatosis) and the subsequent onset of NASH [5–8]. However, growing evidence suggests that simple steatosis and NASH are two separate diseases. In this “multiple parallel hit” hypothesis, the accumulated lipotoxic/proinflammatory lipid species interact with other proinflammatory factors to promote the progression to NASH, whereas in other cases, the liver develops steatosis and remains free of inflammation [9–11]. Therefore, various factors, including toxic lipids, nutrients, and other macrophage- and adipose-derived signals, may induce inflammatory insults.

Recent studies suggest that the types of fat alter body weight and steatosis [12, 13].. In mice, saturated fat-stimulated obesity and hepatic steatosis influence the composition of the gut microbiota [14], whereas dietary fish oil containing large amounts of polyunsaturated fatty acids (PUFAs) of the n-3 family reduced body weight and fatty liver in a model of high-fat diet-induced obesity [15]. In addition, recent studies suggest that the types of fat sensitize hepatocytes to inflammation [12, 13]. It has been reported that a change in the long-chain FA composition via Elovl6 modulates the progress of NASH [16, 17]. It has also been reported that high amounts of dietary PUFAs promote hepatic inflammation [18], whereas the n-3 PUFAs eicosapentaenoic acid (EPA, C20:5) and docosahexaenoic acid (DHA, C22:6) reduced NASH pathologies, but EPA and DHA exhibit different effects in preventing atherogenic high-fat diet-induced NASH [19].

TFAs, or *trans* fats, are unsaturated fatty acids (FAs) with at least one or more double bonds in the trans position. TFAs are produced when food manufacturers add hydrogen to saturate or partially saturate the unsaturated bonds of vegetable oils for cooking, frying, or baking. Double bonds in the FA portions of the oils are saturated with hydrogen and rearranged to produce many isomers, which leads to the formation of TFAs [20]. The elevated intake of TFAs has been correlated to an increased incidence of coronary atherosclerotic diseases [21]. With regard to NASH, dietary intake of TFAs induces fat accumulation in the liver [22, 23]. TFAs are clearly hazardous to human health; thus, the Joint WHO/FAO Expert Consultation on Diet, Nutrition, and the Prevention of Chronic Diseases (JECFA) has recommended that the intake of TFAs should be reduced to less than 1% of the energy intake [24]. Hence, TFA substitutes have been developed. However, the health benefits of reducing dietary TFAs are not fully understood. Two practical options exist to replace dietary TFAs: the use of a natural saturated fat without cholesterol, such as palm oil or its fractions, or a newly developed fat hardened by interesterification. Both types of fat have been the subject of nutritional scrutiny over the last 40 years, and both have positive and negative attributes [25]. In any case, with the use of a TFA substitute, the proportion of saturated fatty acids (SFAs) increased, but the benefits and/or risks associated with the use of TFA substitutes have not been evaluated.

It is well established that a choline-deficient, methionine-lowered, L-amino acid-defined diet (CDAA) induces changes mimicking human NASH in Fischer 344 rats, such as steatohepatitis, hepatic fibrosis, liver cirrhosis, and hepatocellular carcinoma, but has only minimal effects on body weight and glucose metabolism in contrast to semi-purified methionine- and choline-deficient diets [26, 27]. Mice were largely resistant to CDAA [28], but Matsumoto et al. and Chiba et al. recently developed a modified CDAA with reduced methionine and an increased amount of fat by the addition of lard (CDAHFD), which effectively induced NASH in mice [29, 30].

In this context, the aim of the present study is to comparably investigate the effects of shortenings with and without TFAs on NASH induced in mice by feeding our original high-fat CDAA (CDAA-HF), and to assess the safety concerns associated with the use of TFA substitutes. As shown later, Primex Z®, a TFA substitute shortening used in the present study, contains a large amount of SFAs. It should be noted, the use of SFAs was not intended as a TFA substitute, but rather a commercially available TFA substitute with a large SFA content was used. In general, the food industry produces TFA substitutes that are suitable for cooking, baking, and frying applications. Therefore, a commercial TFA substitute should be appropriate for such a purpose. Primex Z® is produced from palm oil and hydrogenated soybean oil.

## Methods

### Diets

As a control, a standard laboratory control chow (58% carbohydrate, 13% fat, and 29% protein on a caloric basis) was obtained from CLEA Japan, Inc. (Tokyo, Japan). As experimental diets, CDAA-HF-T(+) (fat content of 45 kcal% by shortening with TFAs, Primex®, and methionine content of 0.1%; ID A16032901) and CDAA-HF-T(−) (fat content of 45 kcal% by shortening without TFAs, Primex Z®, and methionine content of 0.1%; ID A16032902) were made-to-order products from Research Diet Inc. (New Brunswick, NJ, USA). The components of each diet are shown in Additional file 1. It should be emphasized that the contents of CDAA-HF differed from those of CDAHFD and CDAA. Notably, the fat and methionine contents of CDAA were 31 kcal% and 0.17 [26, 27], whereas those of CDAHFD were 60 kcal% and 0.1 [29], respectively. The diets were frozen until use and changed every other day to prevent the formation of oxidized products.

### Animals

Five-week-old male C57BL/6 J mice were purchased from Japan SLC (Shizuoka, Japan) and adapted to the environment for 1 week prior to the study. Mice were housed under temperature-controlled conditions (22 °C on average) in colony cages under a 12-h light/12-h dark cycle with ad libitum access to food and water. At 6 weeks of age, mice were randomly assigned to one of three groups that were fed the control chow, CDAA-HF-T(+), or CDAA-HF-T(−) for 13 (*n* = 4–5) or 26 (*n* = 10–11) weeks during which body weight, food consumption, and water intake were monitored weekly. At the end of the experimental periods, blood samples were collected from the abdominal aorta of all mice in a non-fasting state, and mice were sacrificed by exsanguination under light isoflurane anesthesia in the early light phase. During autopsy, all organs were carefully observed, and the liver and organs with lesions were excised and weighed.

### Histopathological analysis

Liver samples were fixed in 10% neutrally buffered formalin, embedded in paraffin, and cut into 4 μm-thick sections for hematoxylin–eosin and Sirius Red staining. For Sirius Red staining, the area of fibrosis was measured using cellSens Dimension software (Olympus, Tokyo, Japan) by a scientist blinded to the treatment regimen. The histopathological evaluation of hepatocellular proliferative lesions was performed according to the International Harmonization of Nomenclature and Diagnostic criteria were as follows: regenerative hepatocellular hyperplasia, lesions spanning several hepatic lobules where portal triads and central veins are present, and hepatocellular adenoma, lesions that are greater than several lobules and have no portal triads or central veins. The findings and diagnoses were peer-reviewed by a board-certified toxicologic pathologist who was not one of the co-authors to improve the quality of the pathology data. Immunohistochemical analyses were performed as previously described [31] with samples obtained from mice treated for 13 or 26 weeks using the following primary antibodies: rat anti-mouse monoclonal antibody for F4/80 as a marker of macrophages (1:200; Abcam, Cambridge, UK), rabbit antihuman polyclonal antibody for α-smooth muscle actin (α-SMA) as a marker of activated hepatic stellate cells (1:200; Abcam, Cambridge, UK), and rat anti-mouse monoclonal antibody for cytokeratin 8/18 (CK8/18) as a marker of putative hepatocellular preneoplastic lesions (1:500; Developmental Studies Hybridoma Bank, Iowa, USA). The primary antibodies are listed in Additional file 5. An ApopTag peroxidase in situ apoptosis detection kit was used for TdT-mediated dUTP nick-end labeling (TUNEL) (EMD Millipore Corporation, Billerica, MA, USA). The visualization of antibody binding was performed using a Histofine Simple Stain Kit (Nichirei Corp., Tokyo, Japan) for F4/80 and α-SMA or a VectaStain Elite ABC Kit (Vector Laboratories, Burlingame, CA, USA) for CK8/18. All sections were counterstained with hematoxylin and histopathologically examined in a blinded manner, and the findings were graded from normal (1) to severe (4). The numbers of CK8/18-positive putative hepatocellular preneoplastic lesions consisting of 1, 2, 3, or more cells were counted per 10 light microscopic fields (× 200). The numbers of TUNEL-positive cells were counted per 10 light microscopic fields (× 200).

### Plasma and hepatic chemistries

Plasma was prepared from blood samples to measure triglyceride (TG) and total cholesterol (TC) concentrations, and alanine aminotransferase (ALT) activity using an automatic analyzer (DRI-CHEM; Fujifilm, Tokyo, Japan) or colorimetry test kits purchased from Wako Pure Chemical Industries (Osaka, Japan). Hepatic TG and TC levels were measured as previously described [32].

### FA compositions of diets and livers

The FA composition was quantitatively measured by reversed-phase high-performance liquid chromatography coupled with Fourier transform mass spectrometry (LC/FTMS), as previously reported [33]. Briefly, lipid extraction from samples was performed by bead mill homogenization in 1 mL of methanol. The samples were then mixed by constant shaking (Multi Shaker, Tokyo Rikakikai Co., Ltd., Tokyo, Japan) and centrifuged at 15,000 rpm for 5 min. The obtained supernatant was then collected as the lipid extract. This lipid extract was saponified in 0.5 mol/L potassium hydroxide in ethanol/water (96/4, v/v). The reaction was terminated by adding 1 mol/L hydrochloric acid until the solution became acidic, hexane (100 μL) was added, and the solution was mixed by stirring. The mixture was centrifuged, and the upper layer was collected. After evaporation, the residue was dissolved in 100 ng/mL ^18^O_2_ containing methanol and used for LC/FTMS analysis. LC was performed using an LC-20ADXR ternary pump system equipped with a DGU-20A5R degassing unit, SIL-20 AC autosampler, and CTO-20 AC column oven (Shimadzu Co., Ltd., Kyoto, Japan). The LC system was coupled with an LTQ Orbitrap XL hybrid linear ion trap–Fourier transform mass spectrometer (Thermo Fisher Scientific, Waltham, MA, USA). FTMS detection was conducted in full scan mode at a resolution of 30,000 and a range of 140–600. FAs were detected by obtaining the extracted ion chromatograms of deprotonated ions ([M-H]-) at a mass tolerance of 10 ppm. Instrument control, data acquisition, and data processing were performed using Xcalibur 2.1.0 software (Thermo Fisher Scientific, Waltham, MA, USA).

### RNA extraction and analysis

Total RNA was extracted from the liver using Sepasol reagent (Nacalai Tesque, Kyoto, Japan) and reverse-transcribed using a PrimeScript RT Master Kit (Takara Bio Inc., Shiga, Japan), according to the manufacturers’ instructions. Then, quantitative real-time PCR (qPCR) was performed using SYBR Premix Ex Taq polymerase (Takara Bio Inc. Shiga, Japan) and specific primer sets with a Thermal Cycler Dice Real-Time System Single (Takara Bio Inc. Shiga, Japan). The primer sequences for qPCR in this study are shown in Additional file 2. The mRNA expression levels were normalized to those of cyclophilin mRNA. Portions of the RNA samples were subjected to RNA sequencing (RNA-Seq) and corresponding qPCR analyses. RNA-Seq was performed as previously described [34]. Portions of the 100 ng of total RNA from the livers of the control, CDAA-HF-T(+), and CDAA-HF-T(−) groups (treated for 13 weeks, *n* = 3–4) were used for library preparation. Sequencing libraries were generated using a TruSeq RNA Library Preparation Kit v2 (Illumina Inc., San Diego, CA, USA). Principal component analysis (PCA), differential expression analysis, generation of heat maps with hierarchical clustering of samples, and features and functional annotation analyses using Ingenuity Pathway Analysis (IPA) software (Ingenuity Systems, Qiagen Co., Ltd., CA, USA) were performed as previously described [34].

### Immunoblotting

Immunoblotting was performed as previously described [32]. Aliquots of 50 μg of total protein lysates extracted from the livers were subjected to 10% or 12% sodium dodecyl sulfate–polyacrylamide gel electrophoresis and transferred to polyvinylidene fluoride membranes (Millipore, Darmstadt, Germany). The membranes were probed with anti-glyceraldehyde 3-phosphate dehydrogenase (GAPDH, Santa Cruz Biotechnology, Dallas, USA), cleaved caspase 3, caspase 3, phospho-nuclear factor (NF)κB-p65 (Ser536), NF-κB-p65 and IκB (Cell Signaling Technology, Denver, USA) antibodies followed by horseradish peroxidase (HRP)-conjugated anti-mouse or rabbit IgG secondary antibodies (Cell Signaling Technology, Denver, USA). The primary antibodies are listed in Additional file 5. Immune complexes were visualized using enhanced chemiluminescence (Bio-Rad Laboratories, Hercules, CA, USA).

### Statistical analysis

Values are expressed as the mean ± standard deviation (SD). Analysis of variance followed by the Tukey–Kramer test was used to assess differences among groups. For organ weights/BW (%) and histopathological grading scores not suitable for parametric analysis, the nonparametric Kruskal–Wallis test was performed. Differences were considered significant at *P* < 0.05.

## Results

### Dietary and hepatic FA compositions

Dietary FA compositions are shown in Table 1. Compared with the control chow, CDAA-HF-T(+) and CDAA-HF-T(−) contained higher percentages of saturated and monounsaturated FAs, whereas the proportions of n-6 and n-3 polyunsaturated FAs were reduced. The ratio of saturated FAs was particularly higher in CDAA-HF-T(−) than in CDAA-HF-T(+), mainly due to the increase in palmitic acid (C16:0). However, not surprisingly, CDAA-HF-T(+) contained more TFAs.

**Table 1:** Dietary FA compositions

**Ingredient (%)**	**Control chow**	**CDAA-HF-T(+)**	**CDAA-HF-T(−)**
C14:0	0.5	0.3	0.8
C16:0	11.4	13.2	26.1
C16:1 n-7	0.7	0.1	0.2
C18:0	1.1	6.0	4.2
C18:1 n-7	1.6	10.6	0.9
C18:1 n-7 t	0.1	7.2	0.0
C18:1 n-9	23.2	32.6	43.0
C18:1 n-9 t	0.1	3.4	0.1
C18:2 n-6	52.1	25.0	23.8
C18:2 n-6 tt	N.D.	0.6	0.0
C18:3 n-3	3.4	0.4	0.4
C18:3 n-6	N.D.	0.2	0.1
C20:0	0.3	0.4	0.4
C20:2 n-6	0.0	0.0	0.0
C20:3 n-6	N.D.	N.D.	N.D.
C20:4 n-6	N.D.	0.0	N.D.
C20:5 n-3	1.2	N.D.	N.D.
C22:5 n-3	0.1	N.D.	N.D.
C22:6 n-3	4.2	N.D.	N.D.
Total	100	100	100
**Ingredient (%)**	**Control chow**	**CDAA-HF-T(+)**	**CDAA-HF-T(−)**
SFA	13.2	19.9	31.5
MUFA	25.5	43.3	44.0
n-6 PUFA	52.2	25.1	23.9
n-3 PUFA	8.9	0.4	0.4
TFAs	0.2	11.2	0.2
Total	100	100	100
**Total fat (g/100 g diet)**	**Control chow**	**CDAA-HF-T(+)**	**CDAA-HF-T(−)**
	4.5	24.5	24.5

*ND* not detected

Hepatic FA compositions at the end of week 26 are shown in Table 2. The livers of mice fed CDAA-HF-T(+) significantly contained more *cis*-vaccenic (C18:1 n-7), *trans* vaccenic (C18:1 n-7 t), and elaidic acid (C18:1 n-9 t) than those fed CDAA-HF-T(−). Linolelaidic acid (C18:2 n-6, 9 tt) was only detected in the livers of mice fed CDAA-HF-T(+). Furthermore, the livers of mice fed CDAA-HF-T(−) significantly contained higher amounts of γ-linolenic (C18:3 n-6), dihomo-γ-linolenic (C20:3 n-6), and arachidonic acid (C20:4 n-6) than those of mice fed CDAA-HF-T(+), but the amount of linoleic acid (C18:2 n-6) was similar in the livers of mice fed either CDAA-HF-T(+) or CDAA-HF-T(−).

**Table 2:** Hepatic FA compositions at the end of week 26

Ingredient (ng/mg liver)	Control chow	CDAA-HF-T(+)	CDAA-HF-T(−)
C14:0	120 ± 21	1131 ± 192 ^a^	1021 ± 140 ^a^
C16:0	5423 ± 715	27,503 ± 4477 ^a^	30,389 ± 5932 ^a^
C16:1n-7	933 ± 268	4856 ± 1258 ^a^	4165 ± 587 ^a^
C18:0	1984 ± 417	3671 ± 291 ^a^	4479 ± 1021 ^a^
C18:1n-7	830 ± 207	10,380 ± 1441 ^a^	4237 ± 414 ^a^
C18:1n-7 t	16 ± 5	2246 ± 315 ^a^	96 ± 15 ^a, +^
C18:1n-9	4860 ± 898	66,045 ± 12,407 ^a^	83,732 ± 19,926 ^a^
C18:1n-9 t	13 ± 7	1647 ± 156 ^a^	155 ± 44 ^+^
C18:2n-6	7692 ± 1247	35,212 ± 1832 ^a^	33,790 ± 8871 ^a^
C18:2n-6tt	N.D.	901 ± 63	22 ± 7
C18:3n-3	177 ± 55	154 ± 20	124 ± 40
C18:3n-6	50 ± 32	1393 ± 157 ^a^	2517 ± 73 ^a, +^
C20:0	51 ± 22	169 ± 16 ^a^	224 ± 51 ^a^
C20:2n-6	67 ± 11	433 ± 56 ^a^	354 ± 52 ^a^
C20:3n-6	443 ± 62	1404 ± 89 ^a^	2158 ± 470 ^a, +^
C20:4n-6	2889 ± 434	8668 ± 775 ^a^	12,602 ± 3034 ^a, +^
C20:5n-3	400 ± 91	11 ± 1 ^a^	27 ± 13 ^a^
C22:5n-3	148 ± 26	178 ± 17	219 ± 47 ^a^
C22:6n-3	8754 ± 15	2930 ± 476 ^a^	4184 ± 1178 ^a^
Total	34,850 ± 4687	168,925 ± 20,918 ^a^	184,484 ± 41,347 ^a^

Values are presented as the mean ± SD (*n* = 4).

Significantly different from the values of ^a^control chow or ^+^CDAA-HF-T(+) groups.

*N.D.* Not detected.

### Body and organ weights and plasma and hepatic chemistries

Body and organ weights and plasma and hepatic chemistries at the end of week 13 are shown in Table 3. CDAA-HF, either with or without shortening containing TFAs, decreased body weight. Food consumption was reduced in the CDAA-HF groups as compared with the control group, but there was no significant difference in caloric intake among the three groups (data not shown). In addition, CDAA-HF, either with or without shortening containing TFAs, increased the absolute and relative weights of the liver and eWAT, and enhanced plasma ALT and aspartate transaminase (AST) activities than the control chow. Although not significant, plasma TG and TC levels were decreased, whereas the hepatic levels were markedly elevated in these groups. However, there were no significant differences in these parameters between the CDAA-HF-T(+) and CDAA-HF-T(−) groups.

**Table 3:** Organ weights and plasma and hepatic chemistries at the end of week 13

	Control chow	CDAA-HF-T(+)	CDAA-HF-T(−)
Body weight	26.32 ± 1.71	23.72 ± 1.46^a^	22.94 ± 0.99^a^
Liver (g)	1.36 ± 0.06	1.75 ± 0.30^a^	1.76 ± 0.25^a^
Liver/BW (%)	4.85 ± 0.14	7.38 ± 0.83^a^	7.47 ± 0.86^a^
Kidney (g)	0.36 ± 0.04	0.31 ± 0.06	0.27 ± 0.04^a^
Heart (g)	0.17 ± 0.05	0.15 ± 0.06	0.12 ± 0.02
eWAT (g)	0.43 ± 0.11	0.60 ± 0.07^a^	0.51 ± 0.03
eWAT/BW (%)	1.58 ± 0.40	2.53 ± 0.23^a^	2.19 ± 0.19
TG (mg/dL)	169.13 ± 43.18	106.03 ± 10.51^a^	110.34 ± 10.45^a^
TC (mg/dL)	138.71 ± 22.95	101.46 ± 37.72	91.21 ± 8.75
ALT (IU/L)	23.63 ± 0.81	74.26 ± 12.31^a^	83.58 ± 18.62^a^
AST (IU/L)	127.36 ± 96.80	323.53 ± 187.72	259.67 ± 18.57^a^
Liver TG (mg/g)	6.17 ± 3.33	57.91 ± 25.26^a^	58.93 ± 12.78^a^
Liver TC (mg/g)	2.42 ± 0.49	3.53 ± 0.45^a^	3.21 ± 0.26^a^

Values are presented as the mean ± SD (*n* = 4–5).

*eWAT* Epididymal white adipose tissue, *TC* Total cholesterol, *TG* Triglyceride.

^a^Significantly different from the control value

**Table 4:** Incidences of hepatocellular proliferative lesions at the end of week 26

Lesion	Control chow	CDAA-HF-T(+)	CDAA-HF-T(−)
Foci of cellular alteration	0 (0)	1 (9)	10 (100)
Hyperplasia, hepatocellular, regenerative	0 (0)	1 (9)	3 (30)

*n* = 10–11.

### Nonproliferative liver lesions

**Fig. 1:**
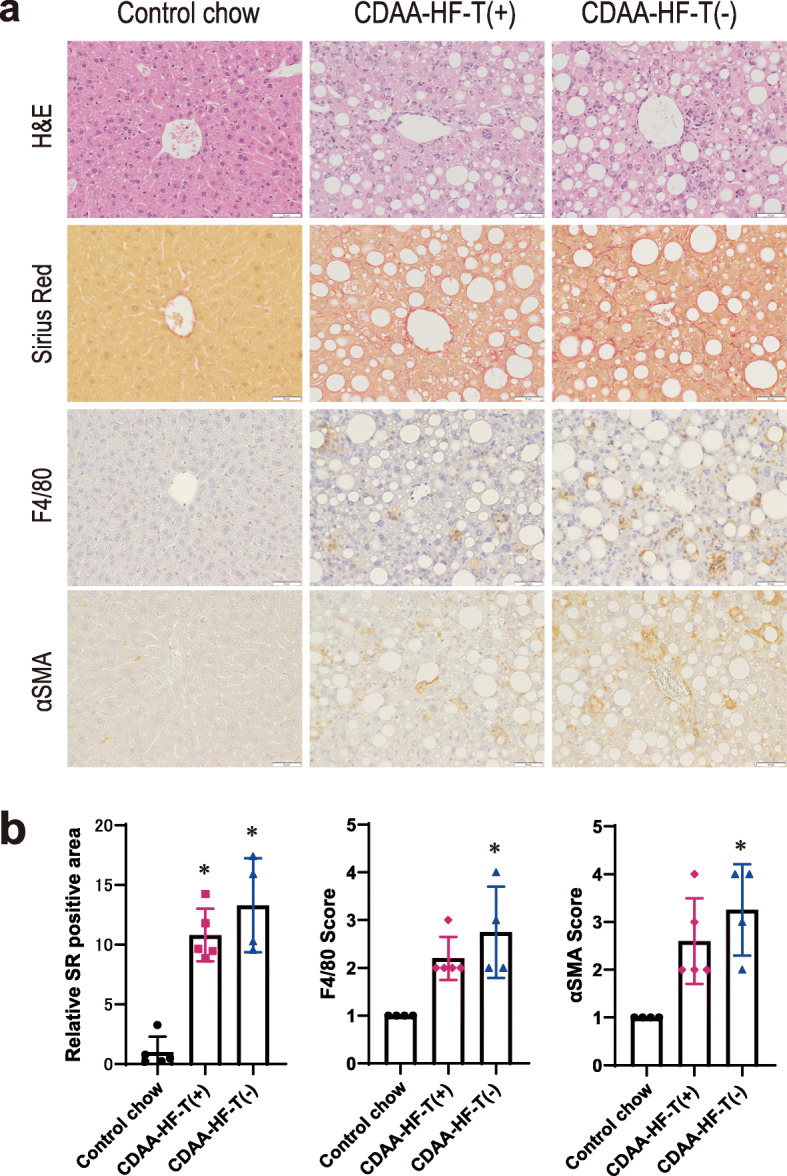
Nonproliferative liver lesions. Histopathological features of hematoxylin–eosin and Sirius Red staining and F4/80- and α-SMA-immunohistochemistry (**a**). Graded from normal (1) to severe (4) (**b**). The following values are presented as the mean + SD. Sirius Red staining: control chow, 1.00 ± 1.29; CDAA-HF-T(+), 10.82 ± 2.21; CDAA-HF-T(−) 13.29 ± 3.94. F4/80: control chow, 1.00 ± 0.00; CDAA-HF-T(+), 2.20 ± 0.45; CDAA-HF-T(−), 2.75 ± 0.96. α-SMA: control chow, 1.00 ± 0.00; CDAA-HF-T(+), 2.60 ± 0.89; CDAA-HF-T(−), 3.25 ± 0.96. *Significantly different from the control value

The representative microscopic features of non-proliferative liver lesions and their gradings are shown in Fig. 1. At the end of week 13, macrovesicular steatosis characterized by hepatocytes with a single cytoplasmic large vacuole was observed in almost all hepatocytes in the CDAA-HF groups. Furthermore, inflammatory clusters where Kupffer cells and hypertrophied macrophages and the number of activated stellate cells were markedly accumulated were observed in these groups, as demonstrated by the immunohistochemical staining of the macrophage marker F4/80 and α-SMA, respectively. In addition, Sirius Red staining revealed fibrosis in the CDAA-HF groups. While the magnitudes of fatty changes and fibrosis were similar within these groups, the inflammation-related changes were only slightly greater in CDAA-HF-T(−) mice than in CDAA-HF-T(+) mice.

At the end of week 26, while steatosis and inflammation remained unchanged, hepatic fibrosis continuously progressed in the CDAA-HF groups (see Additional file 3).

### Proliferative liver lesions at the end of week 26

**Fig. 2:**
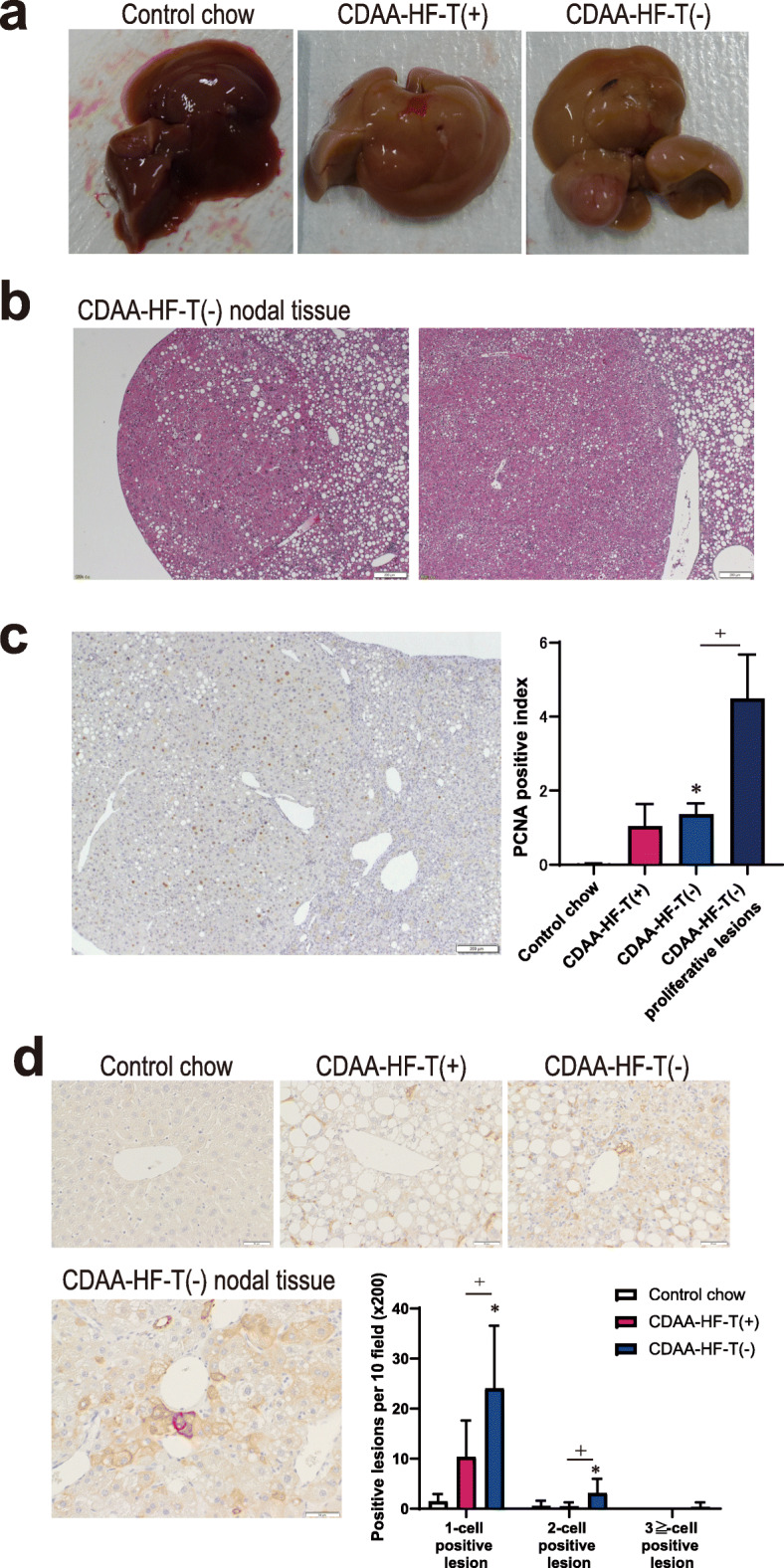
Proliferative liver lesions at the end of week 26. Macroscopic features of the liver from mice (**a**). Microscopic features (hepatocellular hyperplasia and adenoma) of hepatic proliferative lesions of mice fed the CDAA-HF-T(−) diet (**b**). Histopathological features of PCNA staining. PCNA-positive index in normal liver tissue of control animals, nonproliferative liver tissues of the CDAA-HF-T(+) and CDAA-HF-T(−) groups, and proliferative liver lesions of the CDAAHF-T(−) group. The following values are presented as the mean + SD. Control chow, 0.04 ± 0.02; CDAA-HF-T(+), 1.05 ± 0.60; CDAA-HF-T(−), 1.37 ± 0.29; CDAA-HF-T(+), 1.05 ± 0.60; CDAA-HF-T(−) in proliferative lesions, 4.49 ± 1.19 (**c**). The number of CK8/18-positive, putative hepatocellular preneoplastic lesions. The following values are presented as the mean + SD. One-cell positive lesion: control chow, 1.44 ± 1.42; CDAA-HF-T(+), 10.36 ± 7.26; CDAA-HF-T(−), 24.00 ± 12.52. Two-cell positive lesion: control chow, 0.56 ± 1.01; CDAA-HF-T(+), 0.45 ± 0.82; CDAA-HF-T(−), 3.10 ± 2.88. Three-cell positive lesion: control chow, 0.00 ± 0.00; CDAA-HF-T(+), 0.00 ± 0.00; CDAA-HF-T(−), 0.30 ± 0.95. **d** *Significantly different from the control value. Versus chow group; ^+^Significantly different from the CDAA-HF-T(+) value

At the end of week 26 in CDAA-HF groups, rough surfaces and nodules were macroscopically observed in the liver (Fig. 2a), which corresponded to the microscopical identification of hepatocellular proliferative lesions (Fig. 2b). The proliferative characteristics of the lesions were evidenced by the high proliferating cell nuclear antigen (PCNA) -positive index, which were significantly increased in the nonproliferative liver tissues of the CDAA-HF-T(−) group compared with the control group. The abundance of proliferative liver lesions of CDAAHF-T(−) was notably increased (Fig. 2c). The foci of cellular alteration are proliferative lesions, including putatively preneoplastic lesions, which were observed in 1/11 mice (9%) and 10/10 mice (100%) in CDAA-HF-T(+) and CDAA-HF-T(−) groups, respectively (Table 4). Regenerative hepatocellular hyperplasia lesions are proliferative but not preneoplastic [35] and were observed in 1/11 mice (9%) and 3/10 mice (30%) in CDAA-HF-T(+) and CDAA-HF-T(−) groups, respectively (Table 4).

The number of hepatocytes immunohistochemically positive for CK8/18, a marker for preneoplastic hepatocellular lesions [36], was increased in CDAA-HF groups, among which the number was higher in the CDAA-HF-T(−) group than in the CDAA-HF-T(+) group (Fig. 2d).

### Gene expression profiles

**Fig. 3:**
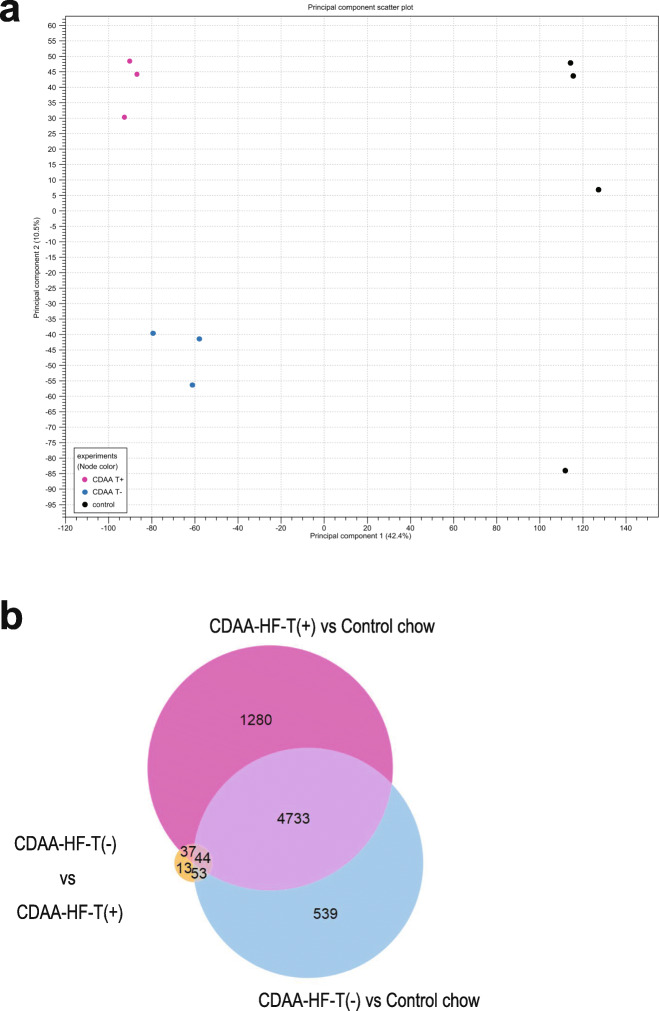
Gene expression profile at the end of week 13. Two-dimensional plot of the principal component analysis for RNA-Seq (**a**). Venn diagram of the comparison of differentially expressed genes (DEGs) based on RNA-Seq data (**b**). Red, CDAA-HF-T(+) vs. control chow; blue, CDAA-HF-T(−) vs. control chow; and yellow, CDAA-HF-T(−) vs. CDAA-HF-T(+)

The RNA sequencing analysis was performed using liver samples obtained at the end of week 13. To identify outlier samples for quality control and determine the primary causes of variation in the dataset, PCA was conducted (Fig. 3a). The control chow and CDAA-HF groups were separated by the first principal component (42.4%, horizontal axis). Then, the second principal component (19.8%, vertical axis) separated CDAA-HF-T(+) and CDAA-HF-T(−) groups. The analyses of various differentially expressed genes (DEGs) were performed between the control chow and either CDAA-HF-T(+) or CDAA-HF-T(−) under the conditions of a false discovery rate (FDR) *P* value < 0.05 and fold change (FC) > ±1.5. The comparison between CDAA-HF-T(+) and CDAA-HF-T(−) groups was conducted with an FDR *P* value < 0.05. As shown by the Venn diagram presented in Fig. 3b, there were a total of 1280 DEGs between the control and CDAA-HF-T(+) groups, 539 between the control and CDAA-HF-T(−) groups, and 13 between the CDAA-HF-T(+) and CDAA-HF-T(−) groups.

### Pathway analysis and hepatic status of apoptosis and NF-κB signaling

**Fig. 4:**
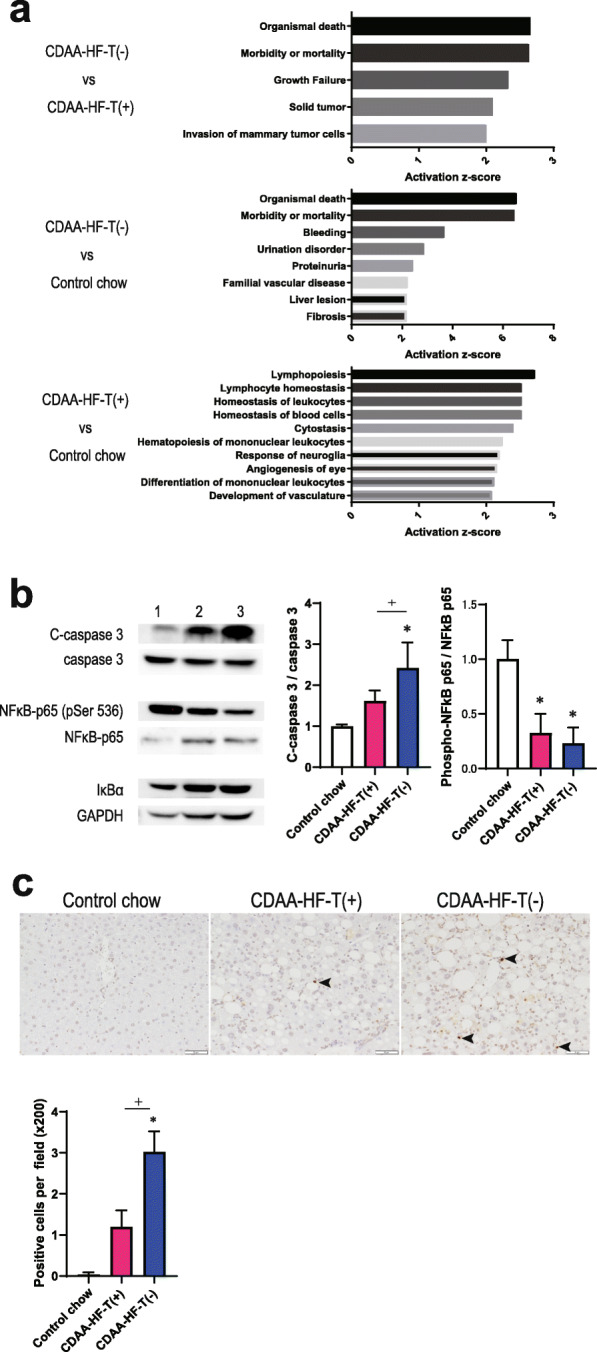
Pathway analysis and hepatic status of apoptosis and NF-κB signaling at the end of week 13. The genes were selected by expression analysis for comparisons between the control, CDAA-HF-T(+), and CDAA-HF-T(−) groups, with an FDR *P* value < 0.05 and/or FC > ±1.5. Activated disease or functional annotation (|z-score| ≥ 2) for DEGs in Ingenuity Pathway Analysis (**a**). Densitometric outcomes of the immunoblot analyses of cleaved caspase 3 and phosphorylated NF-κB-p65 and I-κBα levels expressed as the ratios versus the caspase 3, NF-κB-p65, and GAPDH levels, respectively. The control diet (1), CDAA-HF-T(+) (2), and CDAA-HF-T(−) (3) groups. The following values are presented as the mean + SD. Cleaved caspase 3 / caspase 3: control chow, 1.00 ± 0.05; CDAA-HF-T(+), 1.61 ± 0.26; CDAA-HF-T(−), 2.43 ± 0.61. Phosphorylated NF-κB-p65 / NF-κB-p65: control chow, 1.00 ± 0.17; CDAA-HF-T(+), 0.33 ± 0.17; CDAA-HF-T(−), 0.23 ± 0.14. **b** Histopathological features and the number of TUNEL-positive hepatocytes. The following values are presented as the mean + SD. Control chow, 0.03 ± 0.58; CDAA-HF-T(+), 1.20 ± 0.40; CDAA-HF-T(−), 3.03 ± 0.50 (**c**)

The activation states of diseases and functions were predicted according to the functional analysis of the DEGs using IPA. If there was a change in the data that was consistent with the activation of biological function, IPA presented a predicted z-score, a statistical measure of the correlation between the relationship direction and gene expression predictive of activation (z-score ≥ 2.00) [37]. The activation z-score of ≥2 of CDAA-HF-T(+) versus the control chow, CDAA-HF-T(−) versus the control chow, and CDAA-HF-T(−) versus CDAA-HF-T(+) were shown in Fig. 4a. In the CDAA-HF-T(−) group, genes related to cell death, such as organismal death and mortality, were overexpressed. In contrast, genes related to the immune system, such as lymphopoiesis and the homeostasis of leukocytes, were overexpressed in the CDAA-HF-T(+) group. The selected signaling pathways by IPA analysis were listed in Additional file 4.

Thapaliya et al. found that the expression of active caspase 3 in NASH specimens was strongly correlated with apoptosis in hepatocytes and the progression of NASH [38]. The level of cleaved (activated) caspase 3 protein was significantly increased in the CDAA-HF-T(−) group compared with the control and CDAA-HF-T(+) groups (Fig. 4b).

NF-κB mediates both proinflammatory and antiapoptotic responses, thereby protecting hepatocytes from cell death when inflammatory and immune responses are initiated. Therefore, NF-κB signaling makes an essential contribution to liver homeostasis and wound-healing processes [39]. NF-κB phosphorylation (activation) was greatly attenuated in both CDAA groups (Fig. 4b). Furthermore, the protein level of IκBα tended to increase in the CDAA-HF groups.

The number of TUNEL-positive hepatocytes was higher in the CDAA-HF groups than in the control group at the end of week 13 (Fig. 4c), as well as in the CDAA-HF-T(−) mice compared with the CDAA-HF-T(+) mice.

### Sulfotransferase family 1E member 1 (SULT1E1) and insulin-like growth factor (IGF)-1 expression levels

**Fig. 5:**
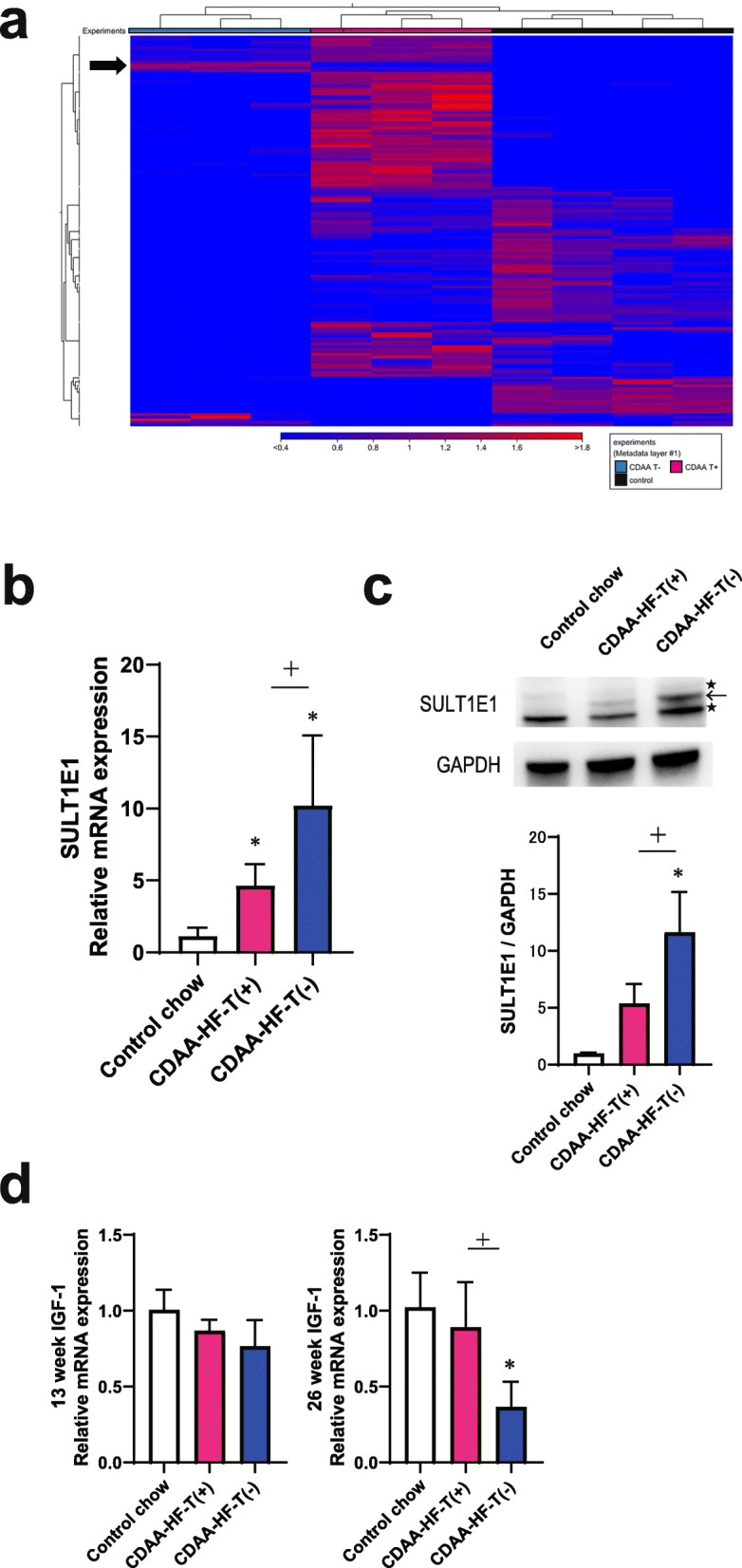
SULT1E1 and IGF-1 expression levels. Two-dimensional heat map of the expression values of DEGs where arrows indicate the genes overexpressed only in the CDAA-HF-T(−) group (**a**). qPCR of the *SULT1E1* gene The following values are presented as the mean + SD. Control chow, 1.14 ± 0.58; CDAA-HF-T(+), 4.65 ± 1.47; CDAA-HF-T(−), 10.20 ± 4.87 (**b**). Immunoblot analysis of the SULT1E1 protein. Densitometric outcomes of the immunoblot analyses of SULT1E1 levels expressed as the ratios versus GAPDH level. The following values are presented as the mean + SD. Control chow, 1.00 ± 0.03; CDAA-HF-T(+), 5.37 ± 1.73; CDAA-HF-T(−), 11.60 ± 3.57. ← indicates SULT1E1 band, ★ stands for non-specific bands (**c**). qPCR of the *IGF-1* gene at the end of weeks 13 and 26. The following values are presented as the mean + SD. *IGF-1* gene at the end of week 13: control chow, 1.01 ± 0.13; CDAA-HF-T(+), 0.87 ± 0.07; CDAA-HF-T(−), 0.77 ± 0.17. *IGF-1* gene at the end of week 26: control chow, 1.02 ± 0.23; CDAA-HF-T(+), 0.89 ± 0.30; CDAA-HF-T(−), 0.37 ± 0.17. **d** *Significantly different from the control value. ^+^Significantly different from the CDAA-HF-T(+) value

Using the genes selected by the expression analysis, the clustering of individual mice (horizontal axis) and information between genes (vertical axis) was conducted to generate a heat map (Fig. 5a). The results indicated that these clusters were divided in the control chow, CDAA-HF-T(+), and CDAA-HF-T(−) groups. According to the heat map, genes overexpressed or downregulated only in the CDAA-HF-T(−) group were further explored. Four genes were identified to be overexpressed in the CDAA-HF-T(−) group. Among them, qPCR revealed that the mRNA expression of the *SULT1E1* gene (Fig. 5b) was markedly increased in the CDAA-HF-T(−) group. In the immunoblot analysis, SULT1E1 protein expression was greatly increased in the CDAA-HF-T(−) group, whereas the magnitude of this change tended to increase, but not significantly, in the CDAA-HF-T(+) group (Fig. 5c). It has been reported that enhanced SULT1E1 activity may play a role in inhibiting growth hormone (GH)-stimulated IGF-1 synthesis via the sulfation and inactivation of β-estradiol (E2) [40]. The *IGF-1* gene tended to be downregulated in the CDAA-HF-T(−) group at the end of week 13, and its expression was further and significantly decreased at the end of week 26 (Fig. 5d).

## Discussion

This study aimed to determine whether TFAs and a substitute have different effects on CDAA-HF diet-induced NASH in mice. While basic outcomes were similar in the livers of mice fed CDAA-HF containing either TFAs or a substitute, the proliferative liver lesions were more substantial in the CDAA-HF-T(−) group than in the CDAA-HF-T(+) group. It is suggested that this increased hepatotoxicity in CDAA-HF-T(−) mice is due to the proapoptotic hepatic microenvironment at a relatively early stage.

The toxic effects of excess lipids, known as lipotoxicity, have recently been identified to cause hepatocellular damage and chronic inflammation in the liver and to be one of the major causes of NASH [13, 41]. The major determinant of lipotoxicity for NASH is not the total amount of TG stored in hepatocytes, but it is the specific class of lipids that damages hepatocytes [13, 41]. Growing evidence suggests that TFAs aggravate nonalcoholic fatty liver disease (NAFLD) and NASH. For instance, oil containing a large amount of TFAs promote liver steatosis and injury through the enhancement of lipid synthesis, hepatocellular necrosis and apoptosis, and cytokine secretion from Kupffer cells [22, 23, 42]. In the present study, while CDAA-HF-T(+) and CDAA-HF-T(−) equally caused the accumulation of hepatic lipid content, inflammatory and fibrotic changes (represented by F4/80 and α-SMA scores, respectively) were only slightly increased in the CDAA-HF-T(−) group than in the CDAA-HF-T(+) group at the end of week 13. At the end of week 26, more hepatocellular proliferative lesions, either preneoplastic or non-neoplastic, had developed in the CDAA-HF-T(−) group than in the CDAA-HF-T(+) group. Therefore, CDAA-HF-T(−) may exhibit more severe toxic and possibly neoplastic effects in the liver of mice than CDAA-HF-T(+), which is likely not simply due to the quantitative difference in dietary or hepatic lipids but to the qualitative difference caused by the absence and presence of TFAs as well as changes in FA profiles.

It has been proposed that hepatic FA composition is an important determinant of NASH progression [16, 17, 43, 44]. Arachidonic acid-derived prostaglandins and related lipid metabolites are active mediators of inflammation [45–47]. In addition to oxidative stress, cyclooxygenase (COX) 2 activity and prostaglandin (PG)E_2_ content are the major factors involved in the mechanisms underlying hepatotoxicity and hepatocarcinogenicity in rats fed CDAA [48]. Compared with the CDAA-HF-T(+) group, the CDAA-HF-T(−) group showed a significant increase in γ-linolenic (C18:3 n-6), dihomo-γ-linolenic (C20:3 n-6), and arachidonic acid (C20:4 n-6) but no change in linoleic acid (C18:2 n-6) in the liver. These results suggest that CDAA-HF-T(−) may promote liver injury by upregulating n-6 PUFA synthesis and metabolism more progressively than CDAA-HF-T(+).

Accumulating evidence indicates that TFAs is a risk factor of NASH in animals [22, 49]. For instance, in low-density lipoprotein (LDL) receptor-knockout weaning male mice fed a 16-week high-fat diet (40% of energy as fat) enriched with TFAs (especially elaidic acid (C18:1 n-9 t)), the development of NASH is more severe. Specifically, more histopathological liver lesions characterized by macrovesicular steatosis and inflammatory cell infiltration were observed compared with mice fed similarly high-fat diets enriched with PUFAs (especially linoleic acid (C18:2 n-6)) or saturated FAs (SFA) (especially palmitic acid (C16:0)), which induced only mild microvesicular hepatic steatosis and minimal inflammation [23]. However, in a recent study using a Western diet mouse model of steatohepatitis, a stronger induction of proinflammatory cytokines and collagen accumulation was observed when non-trans fats were used as a fat source compared with the use of trans fat or corn oil [50]. In addition, Antunes et al. reported that chronic ingestion of Primex-Z, as compared with other common fat sources, including trans-fat, palm oil alone, and corn oil alone, worsened liver injury and enhanced susceptibility to bacterial infections [51]. In the present study, CDAA-HF-T(−) containing a high amount of palmitic acid caused greater NASH-like hepatotoxicity (and possibly hepatocarcinogenicity) than CDAA-HF-T(+) containing TFAs, such as trans vaccenic acid (C18:1 n-7 t) and elaidic acid in association with cis-vaccenic acid (C18:1 n-7). It has been reported that vaccenic acid (C18:1 n-7 t), a predominant ruminant-derived TFAs in the food chain, ameliorates hyperlipidemia and the NAFLD activity score [52]. The Primex Z® used as a fatty source in CDAA-HF-T (−) consisted of palm oil and hydrogenated soybean oil, which may have resulted in an increase in the SFA content. Thus, one possible explanation is that excessive SFAs may be associated with a higher risk of NASH than TFAs. Palmitate itself is known to induce hepatic injury [53]. In the LDL receptor-knockout male mice, CDAA modified by including 1% cholesterol and 41% palm oil, containing a high amount of palmitate, induces NASH and hepatocellular carcinomas within 39 weeks [54]. This indicates that progression of steatohepatitis is not important for particular types of FAs, such as TFAs but is important for comprehensive toxic or protective FAs balance.

RNA sequencing revealed that genes involved in cell death, such as organismal death and mortality, were overexpressed in the CDAA-HF-T(−) group. Cell death, including apoptosis, is essential in the progression of NAFLD and NASH and correlates with progressive inflammation and fibrosis [55]. While both CDAA-HF-T(+) and CDAA-HF-T(−) induced hepatocellular apoptosis, the effect of CDAA-HF-T(−) was more substantial than CDAA-HF-T(+). Similarly, both CDAA-HF-T(+) and CDAA-HF-T(−) reduced NF-κB phosphorylation, but the effect of CDAA-HF-T(−) was greater than CDAA-HF-T(+). Together, these results suggest that the increased hepatotoxicity of CDAA-HF-T(−) is due to the more proapoptotic hepatic microenvironment partially introduced by the inhibition of NF-κB phosphorylation. In fact, Luedde proposed that NF-κB acts as a central link between hepatic injury, fibrosis, and hepatocellular carcinoma and that it may serve as a target for their prevention and treatment [39]. Nevertheless, NF-κB can act as a double-edged sword, and the inhibition of NF-κB may not necessarily exert beneficial effects and can potentially negatively impact on hepatocyte viability [56].

The mRNA expression of the *SULT1E1* gene was specifically increased in the CDAA-HF-T(−) group. In addition, SULT1E1 protein expression was significantly increased in the CDAA-HF-T(−) group by western blotting analysis. The *SULT1E1* gene encodes sulfotransferase family E1 that is responsible for the sulfation and inactivation of E2 [57]. It has previously been reported that SULT1E1 is expressed in hepatocytes, and its activity was significantly elevated in the livers of cystic fibrosis-associated liver disease model mice [58, 59]. These results and those of previous reports suggest that enhanced expression of SULT1E1 may be a cause or result of the events occurring throughout the course of NASH. In either case, this change likely has important implications in the underlying molecular mechanisms of NASH and thus can serve as a potential target to control the disease. However, further studies are required to test these possibilities. The enhanced SULT1E1 activity may also inhibit GH-stimulated STAT5b phosphorylation and IGF-1 synthesis via the sulfation and inactivation of E2 [40]. In fact, the *IGF-1* gene was downregulated in the CDAA-HF-T(−) group. GH and IGF-I coordinately play essential roles in the liver [60]. They are thought to be molecular targets for the treatment of NASH and/or cirrhosis [61–63]. Thus, elevated SULT1E1 may negatively regulate IGF-1 synthesis and thereby GH signaling, contributing to the progression of NASH. Further studies are required to elucidate the detailed pathway or mechanism.

### Study strengths and limitations

In the present study, the mouse NASH model was useful to assess the advanced stage of NASH and thus may be applicable to evaluate inflammation, fibrosis, and neoplastic lesions. Conversely, as a limitation, the model was not appropriate to assess metabolic syndrome. In addition, the elevation in lipids was a result of using a single TFA substitute; therefore, it is necessary to carry out a wide range of studies in the future.

## Conclusions

In conclusion, the replacement of dietary TFAs with a substitute aggravated the development of liver proliferative lesions nutritionally induced NASH in mice, at least under the present conditions. This aggravation may be because the shortening with TFA substitute contained more toxic FAs compared with the shortening with TFAs. Attention should thus be paid regarding future TFA substitute use in humans, and the FA balance is likely more important than the presence of particular types of FAs, such as TFAs and SFAs. On the other hand, it is suggested that the aggravation of NASH in the CDAA-HF-T(−) group may be due to the proapoptotic hepatic microenvironment, introduced by the partial inhibition of the NF-κB phosphorylation and the overexpression of SULTE1E1. These factors can serve as novel molecular targets for the prevention and/or treatment of NASH. The present findings indicate that the replacement of TFAs with a TFA substitute is not necessarily favorable for the promotion of human health. A reduction in TFAs is clearly needed, but caution is required for potential hazards and risks of TFA substitutes. Because only one particular TFA substitute was used in the present study, the data cannot be generalized to all/most TFA substitutes. Thus, additional data should be accumulated using a variety of available TFA substitutes and rigorously scrutinized for their favorable and non-favorable effects on human health.

## Supplementary Information

**Additional file 1:** **Table S1**. Compositions of experimental diets used in this study.

**Additional file 2:** **Table S2**. Sequence information of primers for the quantitative real-time PCR analysis

**Additional file 3.** Nonproliferative histopathological features at the week 26.

**Additional file 4.** Diseases and function analysis of differentially expressed genes by Ingenuity Pathway Analysis (IPA)

**Additional file 5:** **Table S3**. Primary antibody list and specifications

**Additional file 6.**

**Additional file 7.**

## Data Availability

Not applicable.

## Abbreviations

DEG: Differentially expressed genes
FA: Fatty acid
FDR: False discovery rate
GH: Growth hormone
IPA: Ingenuity Pathway Analysis
LDL: Low-density lipoprotein
NAFLD: Non-alcoholic fatty liver disease
PCA: Principal component analysis
SD: Standard deviation
TC: Total cholesterol
TFA: Trans fatty acid
