# Correction to: A trans fatty acid substitute enhanced development of liver proliferative lesions induced in mice by feeding a cholinedeficient, methionine-lowered, L-amino acid-defined, high-fat diet.

**DOI:** 10.1186/s12944-021-01611-9

**Published:** 2022-01-07

**Authors:** Noriko Suzuki-Kemuriyama, Akari Abe, Kinuko Uno, Shuji Ogawa, Atsushi Watanabe, Ryuhei Sano, Megumi Yuki, Katsuhiro Miyajima, Dai Nakae

**Affiliations:** 1 Department of Nutritional Science and Food Safety, Faculty of Applied Bioscience, Tokyo University of Agriculture, 1–1-1, Sakuragaoka, Setagaya, Tokyo, 156–8502 Japan; 2 Department of Nutritional Science and Food Safety, Graduate School of Applied Bioscience, Tokyo University of Agriculture, 1–1-1 Sakuragaoka, Setagaya, Tokyo, 156–8502 Japan; 3 Department of Food and Nutritional Science, Graduate School of Applied Bioscience, Tokyo University of Agriculture, 1–1-1, Sakuragaoka, Setagaya, Tokyo, 156–8502 Japan

**Correction to: Lipids Health Dis 19, 251 (2020).**

10.1186/s12944-020-01423-3

Following publication of the original article [1], the authors noticed that the first name of one of the co-authors Mr. Uno was wrongly printed. The said author name needs to be corrected from “Kiniko Uno” to “Kinuko Uno”. The original article has been updated.
