# Supplementary material for: A trans fatty acid substitute enhanced development of liver proliferative lesions induced in mice by feeding a choline-deficient, methionine-lowered, L-amino acid-defined, high-fat diet
Source: Lipids Health Dis. 2020 Dec 14;19:251. doi: 10.1186/s12944-020-01423-3 (PMC7737357; doi:10.1186/s12944-020-01423-3)
Supplement: Supplementary file 1 — Additional file 1: Table S1. Compositions of experimental diets used in this study. [file 12944_2020_1423_MOESM1_ESM.pdf]

Table S1. Compositions of experimental diets used in this study.

Ingredient (g/kg diet)	CDAA-HF-T(+)	CDAA-HF-T(-)
L-Alanine	5.1	5.1
L-Arginine	12.7	12.7
L-Methionine	0.9	0.9
L-Aspartate	15.8	15.8
L-Cystine	3.7	3.7
L-Glutamate	28.9	28.9
Glycine	6.2	6.2
L-Histidine-HCl-H2O	3.4	3.4
L-Isoleucine	6.1	6.1
L-Leucine	10.5	10.5
L-Lysine-HCl	9.1	9.1
L-Phenylalanine	7.3	7.3
L-Proline	7.6	7.6
L-Serine	7.2	7.2
L-Threonine	4.6	4.6
L-Tryptophan	1.8	1.8
L-Tyrosine	5.7	5.7
L-Valine	6.3	6.3
Sucrose	341	341
Corn Starch	0	0
Maltodextrin	100	100
Cellulose	50	50
Corn Oil	50	50
Primex Z® (101650)	0	172
Primex® (101400)	172	0
Mineral Mix S10001	35	35
Sodium Bicarbonate	4.3	4.3
Vitamin Mix V10001	10	10
Choline Bitrartrate	0	0
Total	905.2	905.2

**% (w/w)**		
Protein	16	16
Carbohydrate	50	50
Fat	25	25

**kcal %**		
Protein	13	13
Carbohydrate	42	42
Fat	45	45
