# Supplementary material for: A trans fatty acid substitute enhanced development of liver proliferative lesions induced in mice by feeding a choline-deficient, methionine-lowered, L-amino acid-defined, high-fat diet
Source: Lipids Health Dis. 2020 Dec 14;19:251. doi: 10.1186/s12944-020-01423-3 (PMC7737357; doi:10.1186/s12944-020-01423-3)
Supplement: Supplementary file 2 — Additional file 2: Table S2. Sequence information of primers for the quantitative real-time PCR analysis [file 12944_2020_1423_MOESM2_ESM.pdf]

Table S2. Sequence information of primers for the quantitative real-time PCR analysis

Gene name	Forward primer (5' to 3')	Reverse primer (5' to 3')
SULT1E1	TCATGTGAAAGCTTGGTGGGA	GGGCTTTCTCTCCAGGAACT
IGF-1	GCTCTTCAGTTCGTGTGTGGAC	CATCTCCAGTCTCCTCAGATC
