# Supplementary material for: A trans fatty acid substitute enhanced development of liver proliferative lesions induced in mice by feeding a choline-deficient, methionine-lowered, L-amino acid-defined, high-fat diet
Source: Lipids Health Dis. 2020 Dec 14;19:251. doi: 10.1186/s12944-020-01423-3 (PMC7737357; doi:10.1186/s12944-020-01423-3)
Supplement: Supplementary file 3 — Additional file 3. Nonproliferative histopathological features at the week 26. [file 12944_2020_1423_MOESM3_ESM.pdf]

Additional Figure

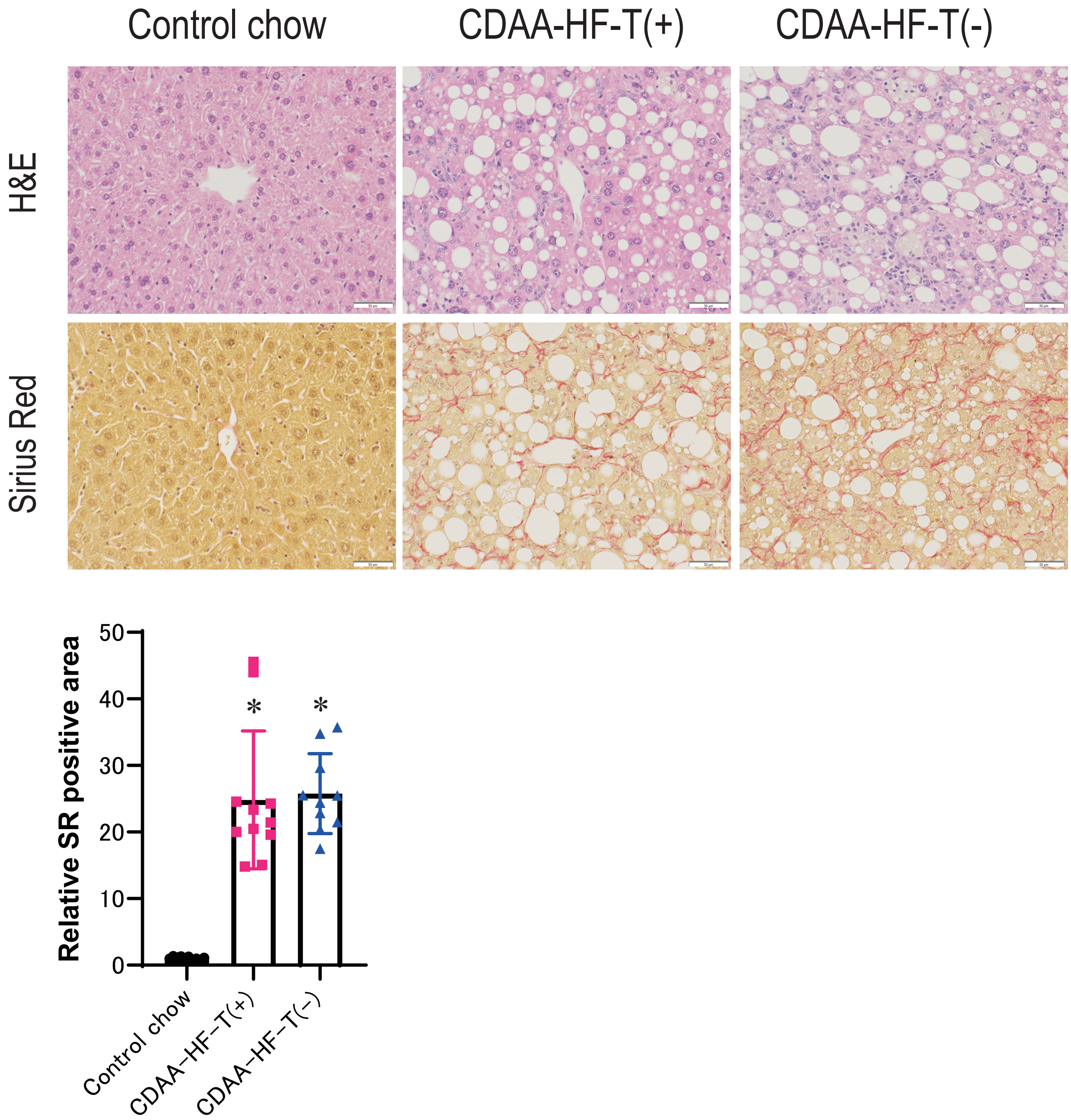

Additional File 3 Nonproliferative histopathological features at the week 26.
Histopathological features of hematoxylin–eosin and Sirius Red staining. The Sirius Red positive area values are presented as the mean + SD. Control chow, 1.00 ± 0.31; CDAA-HF-T(+), 24.83 ± 10.37; CDAA-HF-T(−) 25.77 ± 5.98. *Significantly different from the control value.
