# Supplementary material for: A trans fatty acid substitute enhanced development of liver proliferative lesions induced in mice by feeding a choline-deficient, methionine-lowered, L-amino acid-defined, high-fat diet
Source: Lipids Health Dis. 2020 Dec 14;19:251. doi: 10.1186/s12944-020-01423-3 (PMC7737357; doi:10.1186/s12944-020-01423-3)
Supplement: Supplementary file 5 — Additional file 5: Table S3. Primary antibody list and specifications [file 12944_2020_1423_MOESM5_ESM.pdf]

Table S3. Primary antibody list and specifications.

Antibody	Dilution	Provider	Catalog No.
**Immunohistochemistry**			
F4/80	1:200	Abcam	ab6640
α-SMA	1:200	Abcam	ab5694
CK8/18	1:500	DSHB	TROMA-I
**Immunoblotting**			
P-NFκB-p65 (Ser536)	1:1000	CST	3033
NFκB-p65	1:1000	CST	4764
IκBα	1:1000	CST	4814
Cleaved caspase 3	1:1000	CST	9664
Caspase 3	1:1000	CST	9662
SULT1E1	1:500	Santa Cruz	sc-376009
GAPDH	1:3000	Santa Cruz	sc-25778
