# Supplementary material for: A trans fatty acid substitute enhanced development of liver proliferative lesions induced in mice by feeding a choline-deficient, methionine-lowered, L-amino acid-defined, high-fat diet
Source: Lipids Health Dis. 2020 Dec 14;19:251. doi: 10.1186/s12944-020-01423-3 (PMC7737357; doi:10.1186/s12944-020-01423-3)
Supplement: Supplementary file 7 — Additional file 7. [file 12944_2020_1423_MOESM7_ESM.pdf]

:enago
Author First, Quality First
www.enago.jp

ulatus
localize to globalize
www.ulatus.jp

voxtab
www.voxtab.jp

CERTIFICATE OF EDITING

This is to certify that the paper titled **Enhanced Development of Liver Proliferative Lesions Induced in Mice by Feeding a Choline-Deficient, Methionine-Lowered, L-Amino Acid-Defined, High-Fat Diet** commissioned to us by **Noriko Suzuki-Kemuriyama** has been edited for English language, grammar, punctuation, and spelling by Enago, the editing brand of Crimson Interactive Pvt. Ltd under Advance Editing.

bsi. ISO/IEC 27001 Information Security Management
ISO/IEC 27001:2013 Certified

bsi. ISO 9001 Quality Management
ISO 9001:2015 Certified

Issued by:
Enago, Crimson Interactive Pvt. Ltd.
1001, Techniplex - II, S. V. Road,
Goregaon (W), Mumbai 400062, India.
Phone: 03-5050-5374
Fax: 03-4496-4934

Disclaimer: The author is free to accept or reject our changes in the document after our editing. However, we do not bear responsibility for revisions made to the document after our edit on 29th October, 2020**.**

Global	www.enago.com, www.voxtab.com, www.ulatus.com	Germany	www.enago.de
Japan	www.enago.jp, www.ulatus.jp, www.voxtab.jp	Russia	www.enago.ru
Taiwan	www.enago.tw	Arabic	www.enago.ae
China	www.enago.cn	Turkey	www.enago.com.tr
Brazil	www.enago.com.br	S. Korea	www.enago.co.kr

About Crimson:
Crimson Interactive Inc. provides English language editing, transcription, and translation services to individuals and corporate customers worldwide.

Society for Scholarly Publishing · ISMTE · Council of Science Editors · EASE · American Medical Writers Association · EMWA · BELS
